# Hybridization footprint and the mechanism of leaf color differences in *Philodendron* cultivars

**DOI:** 10.1093/hr/uhag041

**Published:** 2026-02-20

**Authors:** Jiaxuan Chen, Fangping LI, Cong Xu, Jieying Liu, Zhuangwei Hou, Zhilong Huang, Zenpeng Gan, Yuchen Mao, Xiaoran Yan, Haifei Hu, Zefu Wang, Shaokui Wang, HaiPing Fu, Suhong Bu

**Affiliations:** Guangdong Basic Research Center of Excellence for Precise Breeding of Future Crops, Guangdong Provincial Key Laboratory of Plant Molecular Breeding, South China Agricultural University, Guangzhou 510642, China; Institute of Flowers, Agricultural Science Research Center of Dongguan, Dongguan, 523000, China; Guangdong Basic Research Center of Excellence for Precise Breeding of Future Crops, Guangdong Provincial Key Laboratory of Plant Molecular Breeding, South China Agricultural University, Guangzhou 510642, China; Institute of Flowers, Agricultural Science Research Center of Dongguan, Dongguan, 523000, China; Guangdong Basic Research Center of Excellence for Precise Breeding of Future Crops, Guangdong Provincial Key Laboratory of Plant Molecular Breeding, South China Agricultural University, Guangzhou 510642, China; Guangdong Basic Research Center of Excellence for Precise Breeding of Future Crops, Guangdong Provincial Key Laboratory of Plant Molecular Breeding, South China Agricultural University, Guangzhou 510642, China; Guangdong Basic Research Center of Excellence for Precise Breeding of Future Crops, Guangdong Provincial Key Laboratory of Plant Molecular Breeding, South China Agricultural University, Guangzhou 510642, China; Guangdong Basic Research Center of Excellence for Precise Breeding of Future Crops, Guangdong Provincial Key Laboratory of Plant Molecular Breeding, South China Agricultural University, Guangzhou 510642, China; Guangdong Basic Research Center of Excellence for Precise Breeding of Future Crops, Guangdong Provincial Key Laboratory of Plant Molecular Breeding, South China Agricultural University, Guangzhou 510642, China; Guangdong Basic Research Center of Excellence for Precise Breeding of Future Crops, Guangdong Provincial Key Laboratory of Plant Molecular Breeding, South China Agricultural University, Guangzhou 510642, China; Guangdong Basic Research Center of Excellence for Precise Breeding of Future Crops, Guangdong Provincial Key Laboratory of Plant Molecular Breeding, South China Agricultural University, Guangzhou 510642, China; State Key Laboratory of Tree Genetics and Breeding, Co-Innovation Center for Sustainable Forestry in Southern China, College of Ecology and Environment, Nanjing Forestry University, Nanjing 210037, China; Guangdong Basic Research Center of Excellence for Precise Breeding of Future Crops, Guangdong Provincial Key Laboratory of Plant Molecular Breeding, South China Agricultural University, Guangzhou 510642, China; Institute of Flowers, Agricultural Science Research Center of Dongguan, Dongguan, 523000, China; Guangdong Basic Research Center of Excellence for Precise Breeding of Future Crops, Guangdong Provincial Key Laboratory of Plant Molecular Breeding, South China Agricultural University, Guangzhou 510642, China

## Abstract

The genus *Philodendron* exhibits exceptional diversity and ornamental value, but the genetic and evolutionary mechanisms driving its speciation and trait variation remain largely unknown. In this study, we constructed a haplotype-resolved, near-complete genome of *Philodendron tatei* to investigate its evolutionary origins, resolve its phylogenomic placement within Araceae, reconstruct karyotype evolution, and explore genetic clusters and hybridization patterns within *Philodendron* cultivars. Additionally, the genetic and regulatory mechanisms underlying leaf color variation, a key horticultural trait, were explored. Phylogenomic analysis placed *Philodendron* within the Araceae family and provided insights into its karyotype evolution. Comparative genomic analyses identified five major genetic clusters across the genus, highlighting extensive hybridization and allele-specific expression as key contributors to *Philodendron*’s diversity. To investigate leaf color variation, variant mining and transcriptome profiling were conducted on samples with diverse pigmentation. Functional validation identified *PtSGR1* as a critical regulator of pigmentation formation, with differences in promoter activity driving variation in leaf coloration. Overall, this study provides a comprehensive genomic framework for understanding *Philodendron* evolution and diversity, tracing the significant role of hybridization in shaping its speciation and identifying key genetic mechanisms underlying ornamental traits. These insights advance our understanding of plant evolution, contribute to horticultural innovation, and enhance the genetic resources available for studying this ecologically and economically important genus.

## Introduction

*Philodendron*, a widely recognized genus of tropical ornamental plants in the family Araceae, with more than 400 species [1]. In addition to species diversity (second only to Anthurium) and ecological diversity, *Philodendron* also holds a prominent position in the global horticultural market due to its exceptional ornamental appeal and ease of maintenance [2, 3]. Its popularity as a foliage plant has made it one of the core categories in the horticultural industry. In North America alone, the annual industrial output value associated with *Philodendron* has surpassed $1.2 billion in recent years. This economic value is supported not only by its aesthetic features but also by the unique biological properties of the plant, which have attracted scientific interest. For example, studies have demonstrated that *Philodendron* leaves are rich in chlorogenic acid, a compound known for its potent antioxidant activity [4]. These unique characteristics highlight the dual significance of *Philodendron* as a highly marketable ornamental plant and as a subject of scientific research with potential applications in medicine, horticulture, and beyond.

Given that there are many species in the genus *Philodendron*, taxonomic research remains fragmentary. Previous classifications mainly relied on DNA nuclear markers including internal transcribed spacer (ITS), external transcribed spacer (ETS) and the chloroplast intron *rpl16*, etc. [1, 5, 6]. However, the highly conserved nature of plastid genomes provides limited information and cannot fully capture the complex patterns of hybridization and genetic diversity within species [7]. In recent years, the release of numerous pan-genomic studies (e.g. potato and grapevine) and high-quality reference genomes of polyploid plants with highly heterozygous (e.g. *Phyllanthus emblica*) has provided a new generation of high-throughput methods for taxonomy, while making it feasible to explore the genetic and evolutionary basis of key traits [8–11]. Despite the significant ornamental and economic value of *Philodendron* within the Araceae family, genomic studies on this genus have been limited. So far, only a few high-quality genomes of the Araceae family have been released, including *Acorus tatarinowii* in the Proto araceae subfamily, *Spirodela polyrhiza* in the Lemnoideae subfamily, and *Colocasia esculenta* in the Aroideae subfamily [12–14]. However, there have been no published reference genomes for the genus *Philodendron*. Beyond *Philodendron* itself, genomic data from this genus can contribute to comparative genomics studies within the broader Araceae family, offering insights into karyotype evolution and phylogenetic relationships.

One of the defining traits of *Philodendron* species is their remarkable diversity in morphological features, particularly the form and color of their leaves. These traits greatly enhance their value as foliage plants, with leaf color being a key factor in determining their economic worth. Leaf coloration is influenced by both genetic differences among species and environmental factors. This variation in leaf traits not only contributes to the aesthetic appeal of *Philodendron* but also provides opportunities for developing new cultivars with desirable characteristics. In previous studies, flowering of the genus *Philodendron* was difficult and required measures such as hormone induction [15]. As a result, propagation within the industry primarily relies on *in vitro* tissue culture, and seeds are rarely observed in commercial production systems [16].

In this study, we leveraged multiple sequencing strategies to generate a haplotype-resolved, near-complete genome assembly of *P. tatei*. This high-quality genome assembly allowed us to identify significant differences in k-mer and long terminal repeat (LTR) sequences between the two genomic haplotypes, suggesting that *P. tatei* may have originated from hybrid originate via distant hybridization. Using this genomic data, we determined the evolutionary position of *Philodendron* within the Araceae family and reconstructed the karyotype evolution relationships among species in this family. Additionally, we conducted whole-genome sequencing and comparative analysis of multiple species within the genus *Philodendron*, uncovering five major genetic clusters and further elaborating on the hybridization patterns among genetic clusters. These findings suggestted that the current diversity of *Philodendron* species has been shaped by extensive hybridization and gene introgression.

To further explore the genetic basis of leaf color variation, we performed variant mining and transcriptome analysis on *Philodendron* samples with diverse leaf coloration. This analysis led to the identification of key gene *PtSGR1*, which is involved in regulating leaf color with promoting the degradation of chlorophyll. Plant transformation experiments and promoter activity assays confirmed that differences in the promoter activity of *PtSGR1* play a critical role in leaf color variation among *Philodendron* species. These findings not only provide new insights into the genetic mechanisms underlying leaf trait diversity but also open up possibilities for the development of novel *Philodendron* cultivars with enhanced ornamental value. Such advancements underscore the potential of genomic research to drive innovation in horticulture, while also contributing to our broader understanding of plant biology and evolution.

## Result

### Highly heterozygous genome assembly of the *P. tatei* reveals its reproductive preference characteristics

**Figure 1:**
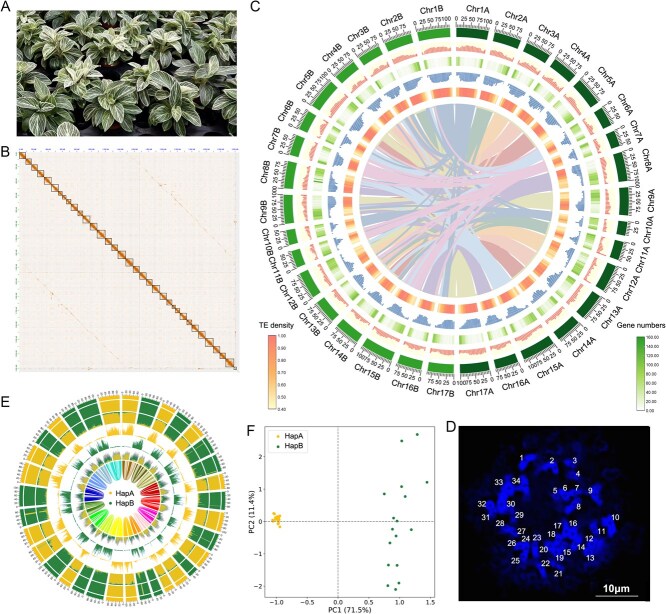
Genomic characteristics of *Philodendron tatei.* (A) Morphology of *P. tatei.* (B) The Hi-C interaction signal of the haplotype-resolved genome assembly. (C) Circos plot of gene features across the 34 chromosomes between two haplotypes of *P. tatei*. From outer to inner circles: GC content, gene density, long terminal repeat (LTR) density, TE density and collinearity. (D) The chromosome karyotype experiment based on root tip preparations of *P. tatei*, the chromosome was marked with numbers. (E) The classification of two types of k-mer among the haplotypes, from outer to inner circles haplotype classification, k-mer enrichment, k-mer proportion, k-mer density, LTR-RTs density and homologous relationships. (F): The PCA of two types of k-mer among the haplotypes.

Understanding the basic characteristics of the genome is extremely important for genome assembly. We selected the mainstream *P. tatei* (Phs20) for genome sequencing and assembly (Fig. 1A). Flow cytometry estimated a genome size of ~1.5 Gb (1C), whereas a k-mer–based genome survey indicated a more homozygous genome size of ~3.0 Gb, consistent with a properly resolved 2C genome showing a unique peak (Supplementary Fig. S1). Under these circumstances, we assembled the *P. tatei* genome using 96 Gb of HiFi data and 53.8 Gb of ONT data, and found that the Primary collapsed consensus contig (Pctg) size of the assembly was approximately 3.05 Gb (N50 = 94.07 Mb) (Supplementary Table S1), corresponding to the characteristics of a 2C genome. Further analysis with Hi-C data revealed that the *P. tatei* genome could be divided into 34 pseudochromosomes, which corresponds with our karyotype assays (2*n* = 2*x* = 34) and the chromosome number range reported in previous studies of *Philodendron* (Fig. 1B–D) [17]. Among them, 28 chromosomes were assembled to a gap-free level. This assembly included 60 complete centromeres, with the entire haplotype A achieving a fully gap-free status. (Supplementary Table S2).

The quality analysis of the genome based on Benchmarking Universal Single-Copy Orthologs (BUSCO) revealed that 97.6% of the single-copy orthologues were completely identified in the assembly (Supplementary Table S3). To assess the quality and completeness of the assembly, the HiFi mapping rate, LTR Assembly Index (LAI), and QV value were evaluated. The results (99.86%, 19.3, and 58.3, respectively) indicate a high level of assembly quality and integrity. Synteny analyses showed that the 34 chromosomes formed two groups (*n* = 17) with relatively weak intergroup synteny; each group had a genome size of ~1.50 and ~1.48 Gb (1C), respectively. Through Subphaser [18], we identified the chromosomes belonging to the A and B haplotypes based on the k-mer distribution characteristics of the genomic DNA sequence of *P. tatei*, and assigned the homologous chromosomes to their respective haplotypes (Fig. 1E). The principal component analysis (PCA) analysis based on k-means also revealed that the chromosomes could be clearly divided into two distinct clusters (Fig. 1F). These two groups of chromosomes have a pairwise linear relationship (Fig. 1C). Further annotations indicate that the transposable element (TE) sequence accounts for approximately 75.83% (~2.26 Gb) of the genome, including 1.88 Gb of LTR sequences (Supplementary Table S4).

The assembly and classification of two haplotypes suggest that the *P. tatei* genome likely originated via allopolyploid hybridization, characterized by high heterozygosity that prevents consistent genome assembly and necessitates haplotype-resolved assembly. Divergence between homologous chromosomes is known to interfere with meiotic pairing and can reduce fertility, which may partially explain why sexual reproduction in *P. tatei* is rarely reported under natural conditions. Gene annotation identified 59 649 genes distributed across 34 chromosomes, and >90% received primary functional annotations. GO enrichment analysis of haplotype-private genes showed that HapA was significantly enriched in transcription factor binding and RNA processing, whereas HapB was enriched in DNA metabolic processes and meiotic cell-cycle functions, indicating functional divergence between haplotypes (Supplementary Fig. S3).

### Evolutionary characteristics of the genus *Philodendron* and karyological evolution of the Araceae family

**Figure 2:**
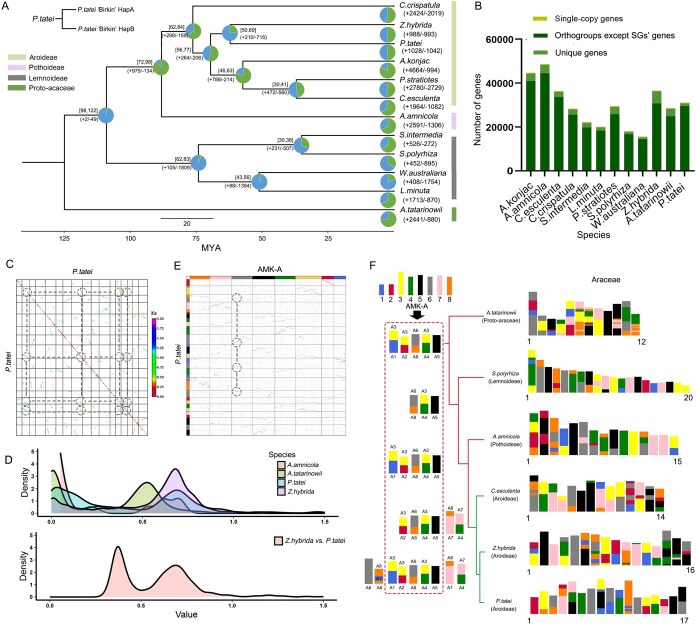
Evolutionary characteristics of the genus *Philodendron* and karyological evolution of the Araceae family. (A) The phylogenetic relationship between *P. tatei* and other Araceae species. (B) The distribution of gene in homologous genes cluster. (C) The orthologous gene pairs and *Ks* distribution in *P. tatei* genome. (D) The *ks* distribution of Araceae species. (E) Ancestral karyotype mapping based on orthologous genes pairs between *P. tatei* and AMK-A. (F) Reconstruction of ancestral karyotypes of various plants in the Araaceae family and identification of the distribution of common ancestor chromosome recombination.

Phylogenetic placement provides essential context for interpreting genome evolution. Using 469 strict single-copy orthologous gene pairs from 12 species (with *A. tatarinowii* as the outgroup), phylogenetic analyses placed *Philodendron* within the Aroideae subfamily of Araceae and identified *Zantedeschia* as its sister genus (Fig. 2A and B). Divergence between these two genera occurred ~50–69 million years ago, during which commonly shared 210 gene families expanded and 716 contracted (Fig. 2A). Expanded gene families in *P. tatei* were significantly enriched for pathways associated with enzymatic activity, consistent with enhanced adaptive potential in complex environments (Supplementary Fig. S4).

The Araceae family is divided into several subfamilies, among which the Aroideae subfamily is the most representative. The identification of homologous gene pairs revealed that there are four pairs of paralogous relationships between the internal chromosomes of *P. tatei* (Fig. 2C). Synonymous substitution rate (*Ks)* distributions for these paralogs showed a single prominent peak around *Ks* ≈ 0.7 (Fig. 2D), a signal shared by multiple true Araceae species. In contrast, the basal Araceae species *Anthurium amnicola* exhibited two peaks near *K*s ≈ 0.7. Interspecies *Ks* comparisons across Araceae formed two peaks (≈0.4 and ≈0.7), corresponding to two sets of orthologous relationships and indicating unequal differentiation among duplicated gene sets. The nuclear evolution of the *P. tatei* species, based on ancestral monocot karyotype except for Acoraceae (AMK-A) [19] with multiple Aroideae species, also indicates that most ancestral chromosomes have four relatively complete copies in these modern species. This suggests that these species may have undergone two common whole genome duplications (WGDs). The formation of two independent *Ks* peaks in orthologues tends to indicate the occurrence of heteromorphic hybridization, which is also a common cause ofpolyploidy.

Species belonging to the Araceae family all have a complete nuclear evolutionary relationship with AMK-A (Fig. 2E and F). Based on the ancestral karyotype remodeling of modern species of the Araceae family, we have detected that the recombination of ancestral chromosomes is widely involved in the development and differentiation processes of these species. Certain ancestral chromosome combinations (A4 + A3 and A6 + A8) were broadly conserved across species, whereas combinations such as A7 + A4 and A8 + A7 were specific to the Aroideae clade. In the case of *P. tatei* and its sister species *Z. hybrida*, there are nine identical ancestral chromosome combinations. It is worth noting that the ancestral chromosome A5 is almost always present in at least one of the modern chromosomes of all species of the Araceae family, remaining completely intact (Fig. 2F; Supplementary Table S5).

### Genetic diversity and hybridization in *Philodendron* species and cultivars

**Figure 3:**
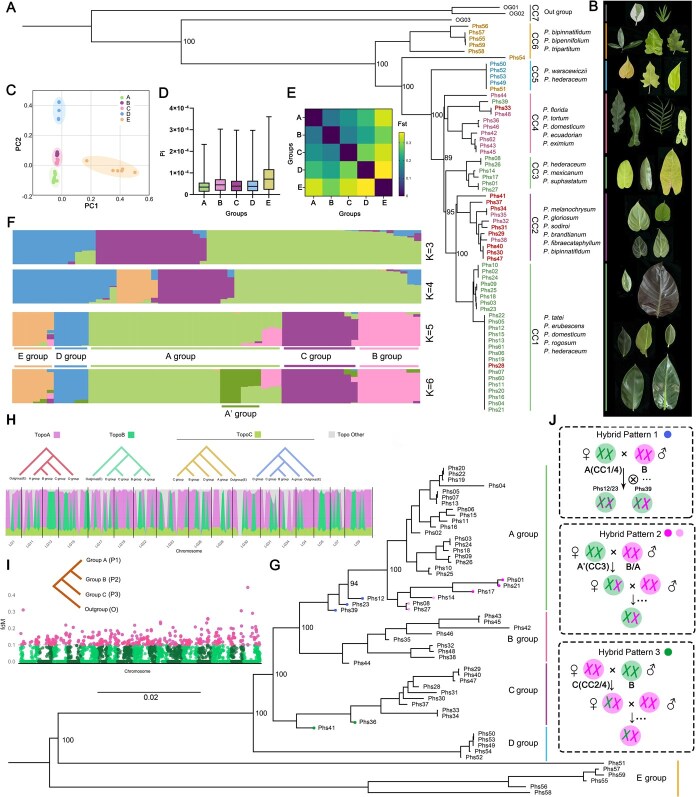
Extensive multi-round hybridization promotes the diversity of *Philodendron* cultivars. (A) The phylogenetic relationship of the plastid system based on chloroplasts genome assembly in *Philodendron* cultivars; two individuals of *Amydrium* (OG1/OG2) and one of *Thaumatophyllum* (OG3) were utilized as outgroups. (B) The leaf phenotypes among various *Philodendron* cultivars. (C) The cluster of PCA with the alignment short read data based on the genome assembly. (D) The Pi nucleic acid diversity of each cluster. (E) The assessment of differentiation among genetic clusters based on Fst. (F) Population genetic structure of *Philodendron* cultivars based on ADMIXTURE analysis. (G) Phylogenetic relationships with ML Tree of based on the SNPs among *Philodendron* cultivars. (H) The topological structure of the phylogenetic tree within *Philodendron* species. (I) The genetic introgression analysis from Group C (P3) to Group B (P2). (J) The possible hybridization patterns within *Philodendron* species population. Hybrid pattern 1: Maternal lineage and nuclear genome from A with B genetic signal; Hybrid pattern 2: Maternal lineage from A’ and nuclear genome from A/B; Hybrid pattern 3: Maternal lineage from C and nuclear genome from B.

To elucidate the genetic underpinnings of diversity within this genus, we sequenced 62 cultivars spanning 23 species (Supplementary Table S6). Phylogenetic inference using a maximum likelihood (ML) tree based on chloroplast genomes showed that these cultivars can be grouped into six chloroplast clusters (CC1–CC6), each exhibiting shared leaf morphological characteristics (Fig. 3A and B). Specifically, CC1 and CC3 groups feature broad or elongated leaves, with stems and leaves often displaying deep red or dark green tones. CC4 group has diverged from CC1 group, exhibiting narrower leaf shapes and heteromorphic leaves, with individual leaf colors being more diverse. CC2 group predominantly displays heart-shaped leaves, often accompanied by velvety textures or light-colored veins. CC5 is characterized by lighter colored individuals, often bearing partially lobed leaves, whereas CC6 mainly includes lobed or palmate leaves.

PCA of nuclear genome SNPs further resolved all individuals into five distinct genetic groups (Group A–E) (Supplementary Fig. S5; Fig. 3C). Population structure analysis supported *K* = 5 as the optimal number of genetic clusters (Fig. 3F; Supplementary Fig. S6). The chloroplast clades largely corresponded to these nuclear groups: CC1/CC3 to Group A, CC4 to Group B, CC2 to Group C, CC5 to Group D, and CC6 to Group E (Fig. 3A and G). Among these, Group E was highly divergent, while Group B occupied an intermediate genetic position between Groups A and C (Fig. 3D and E). Although the Phs20 genome is highly heterozygous, its placement in both the haplotype-based phylogeny and the chloroplast tree within the core *Philodendron* group indicates that its two haplotypes are probably not products of recent hybridization (Fig. 3G; Supplementary Fig. S7).

To investigate evolutionary relationships among groups, we analyzed genome-wide phylogenetic topologies, identifying three predominant topological patterns: TopoA (matching the species tree, Fig. 3G), TopoB (where Groups A/B and Groups C/D each form a clade), and TopoC (where Groups B and C form a clade) (Fig. 3H). TopoA was the dominant pattern and alternated along the chromosomes with TopoB, whereas TopoC, though less frequent, was broadly distributed across the genome. Introgression analysis detected widespread genomic regions with high fdM values (>0.1) from Group C (P3) to Group B (P2), supporting the view that the genome-wide presence of TopoC reflects historical gene flow (Fig. 3I; Supplementary Fig. S8). Additional introgression signals were observed from group B to group A and from group D to group C, which may contribute to the formation of TopoB (Supplementary Tables S7–S9). However, introgressed segments from group B to group A were potentially enriched for genes involved in response to biotic stress, while those from group D to group C may be associated with genes related to diverse enzymatic activities.

Based on the classification of chloroplast genomes, ADMIXTURE analysis and SNPs, we proposed three different hybridization patterns for several specific individuals (Fig. 3J; Supplementary Table S10; Supplementary Fig. S7). In hybrid pattern 1, both haplotypes of Phs12 and Phs23 belong to Group A and their chloroplast genomes are assigned to CC1, yet ADMIXTURE analysis reveals genetic contributions from Group B, suggesting they are selfed progeny of F_1_ hybrids. Phs39 differs: its two haplotypes are basal to Groups A and B, respectively, indicating it is likely an F_1_ hybrid between a selfed progeny of a Group A × Group B cross and a pure Group B individual. Hybrid pattern 2 gives rise to individuals displaying special leaf phenotypes (Phs01, 21, 17 and 14), including heart-shaped (Phs14) and narrow leaves (Phs27 and 08), which align closely with Group B characteristics (Supplementary Fig. S9). Two individuals with nucleo-cytoplasmic incompatibility (Phs41 and Phs36) clustered with B in PCA but grouped with the C species in the phylogenetic tree, suggesting evidence of hybridization (Hybrid pattern 3). Phs36 had a CC4 plastid type, indicating B as the maternal lineage, whereas Phs41 retained a CC2 plastid type, suggesting B as the paternal lineage. Additionally, Group A in the plastid tree was divided into two distinct origins (CC1 and CC3), suggesting two maternal lineages (Fig. 3C). These individuals were separately classified into the A’ group when *K* = 6, which also supports the result of hybrids (Fig. 3F).

### Specific gene expression among haplotypes shapes the leaf color differentiation

**Figure 4:**
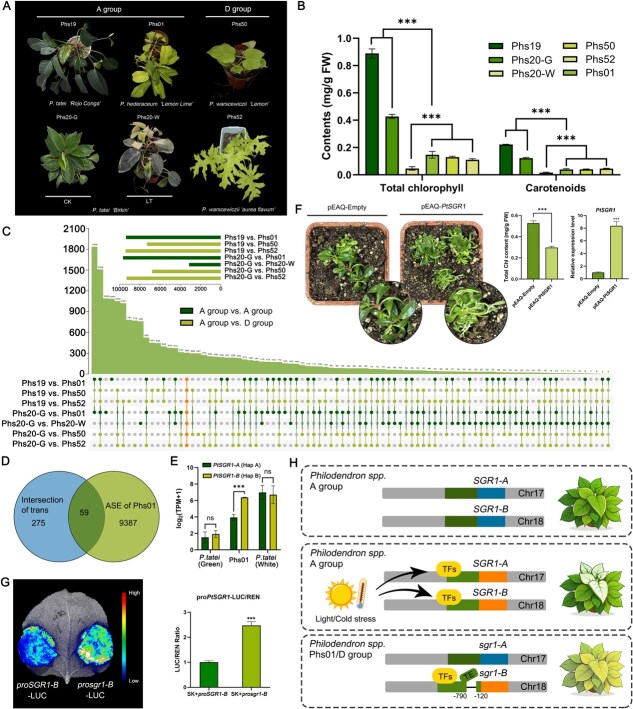
Leaf color differentiation and formation mechanism. (A) Phenotype of leaves among various of *Philodendron* cultivars and *P. tatei* plant in low-temperature (LT) environment (15°C for 30 days). (B) The content of chlorophyll and carotenoids in leaves among various of *Philodendron* cultivars. Data are means ± SE (*n* = 3), ^***^P < 0.01 and ns *P* > 0.05 (Student’s *t*-test), similarly hereinafter. (C) Upset plot of differentially expressed genes (DEGs) generated from the transcriptome based on leaves of *Philodendron* cultivars. (D) The Venn diagram between DEGs’ intersection and Phs01’s ASE. (E) Transcripts Per Million (TPM) of ASEs among *Philodendron* cultivars with different leaf color. (F) The transformation of *PtSGR1* in *P.tatei* leaves leads to chlorosis phenotype and decrease in chlorophyll content. (G) The luciferase assays of different promoters indicate that *sgr1-B* promoter exhibits higher downstream transcriptional activation activity than *SGR1-B*. (H) Differential expression of paired *PtSGR1* alleles (A/B) contributed to the leaf color diversity in *Philodendron* cultivars and species.

As an ornamental plant, *Philodendron* leaves exhibit rich diversity, which directly affect its economic value. The morphological differences in its leaves directly influence its economic value, with leaf color being the most striking among its morphological traits. In population genetics studies, we found that group D (the *P. warszewiczii* branch) exhibits a distinct yellow-leaf trait, while the core *Philodendron* group (A group) also contains cultivars with yellow-leaf traits (Fig. 3B). Through routine phenotypic observations, we detected that many green-leaf *Philodendron* cultivars often display leaf chlorosis (e.g. Phs20-W) under environmental stress such as low temperature and low light (Fig. 4A and B).

To investigate the genetic basis of this trait, we conducted transcriptomic experiments on green-leaf, chlorotic individuals within green-leaf and yellow-leaf cultivars from core *Philodendron*, as well as two yellow-leaf cultivars from group D. Differential expression analysis revealed 190/306 *Philodendron* genes that exhibited consistent upregulated/downregulated expression trends in both green vs. chlorotic leaves and green vs. yellow leaves, respectively (Fig. 4C; Supplementary Fig. S10; Supplementary Table S11). To investigate the association between allele-specific expression (ASE) and leaf color variation in *Philodendron* cultivars, we performed the ASE analysis among three cultivars with different leaf color. We identified a markedly stronger ASE signal in the yellow-leaf cultivar (Phs01), involving about 9000 loci, which were significantly enriched in pathways related to chloroplast morphogenesis (Supplementary Tables S12–S15). Integrating ASE loci with DEGs highlighted a candidate structural gene, *MdrSG200018983* (*PtSGR1-B*), which is implicated in chlorophyll degradation (Fig. 4D and E; Supplementary Table S16). The expression levels of *PtSGR1-B* also showed a significant increase in both yellow-leaf and chlorotic individuals, while *PtSGR1-A* only significantly expressed in chlorotic one (Phs20-W treated with low temperature) (Supplementary Fig. S11). Their homologs, *AtSGR1* in *Arabidopsis thaliana*, has been reported to significantly promote chlorophyll degradation [20, 21]. The coding sequence between *PtSGR1-A/B* revealed no differences, suggesting that the differences between them might be caused by upstream regulatory. To further elucidate the functional role of PtSGR1-B in chlorophyll degradation, transient overexpression assays of *PtSGR1-B* were performed in leaves of *N. benthamiana* and *P. tatei* (Fig. 4F; Supplementary Fig. S12). The results indicated that PtSGR1 significantly promotes leaf chlorosis, suggesting its involvement in the regulation of chlorophyll degradation.

To explore the genetic basis for the differential expression, promoter cloning and ONT-based sequencing of PCR products were performed. This approach identified a ~670 bp deletion in the *PtSGR1-B* promoter of the yellow-leaf cultivar (Phs01, sgr1-B), located approximately 120 bp upstream of the transcription start site (TSS), which is absent in the green-leaf cultivar (Phs20, SGR1-B) (Supplementary Fig. S13). The cis-acting element analysis based on the JASPER database (Supplementary Table S17) revealed that numerous transcription factors (such as MYB, ERF, and Dof) potentially regulate this deleted region, suggesting that variations in the upstream regulatory elements of *PtSGR1-B* may contribute to the differences in leaf coloration among *Philodendron* cultivars. To further investigate differences between the two promoter types, GUS reporter and luciferase/renilla reporter assays were employed in tandem, followed by transformation into *N. benthamiana* leaves to measure promoter activity. Experimental results showed that *sgr1-B* promoter exhibited approximately 2.5 times higher activity than *SGR1-B* promoter, consistent with the expression differences observed among *Philodendron* cultivars (Fig. 4G). At the same time, a stronger generation of X-Gluc hydrolysis products was also observed in *sgr1-B*-transferred *N. benthamiana* in the GUS activity test (Supplementary Fig. S14). In contrast, the promoter of allele *PtSGR1-A* exhibited no sequence variation between Phs20 and Phs01, nor was differential expression observed between yellow and green *Philodendron* cultivars (Supplementary Fig. S11). Together, these results indicate that *PtSGR1-B* promoter variation, in combination with environmental responsiveness, contributes to leaf-color differentiation among *Philodendron* cultivars (Fig. 4H).

## Discussion

The Araceae family is highly regarded in horticulture as a group of ornamental plants due to its unique leaf morphology, vibrant colors, and diverse textures [22]. Among them, *Philodendron* stands out as the second largest ornamental plant group within Araceae, celebrated for its wide variety of leaf shapes and colors. The genome of *Philodendron* is characterized by high heterozygosity and extensive variation. To enable effective sexual breeding in this predominantly clonally propagated species, it is essential to systematically resolve the genomic features of its haplotypes [23]. Cheng *et al.* provided a comprehensive analysis of the potato haplotype pan-genome, unveiling its heterozygosity and haplotype diversity, which offered novel insights into breeding strategies [24]. Despite its significance, the availability of genomic and functional information for *Philodendron* remains limited. Utilizing PacBio and ONT sequencing technologies combined with the Hi-C method, we successfully assembled two haploid-resolved genomes of *Philodendron*, with sizes of 1.50 and 1.48 Gb, respectively. The HapA assembly achieved a gap-free status, presenting the first haplotype-resolved, near-complete genome assembly in *Philodendron*. However, for the highly heterozygous *P. tatei* and other cultivars of *Philodendron*, whose sequences could not be completely collapsed and assembled, the gaps in HapB remain non-negligible and warrant further resolution in future studies. Our findings not only advance the molecular characterization of this economically important genus but also highlight the potential of genomic tools to accelerate breeding programs and comparative genomic studies within the Araceae family.

Building on the assembled genomes, we investigated the evolutionary history and genomic characteristics of *Philodendron*. Phylogenomic analyses revealed that *Philodendron* belongs to the Aroideae clade within the Araceae family, which is part of the core true monocots, consistent with prior phylogenetic studies [25, 26]. WGDs or polyploidization events play a crucial role in plant genome evolution and adaptation to environmental changes [27]. Previous studies have suggested that the rise of core monocots may have been facilitated by the ancestral τWGD event [28]. To further explore this, we performed synteny analyses on *Philodendron* and representative species from the Araceae lineage. The results indicate that *Philodendron* underwent two distinct and widespread WGD events. Additionally, *Ks* distribution analysis for homologous gene pairs revealed the presence of two separate *Ks* peaks, suggesting the occurrence of allopolyploidy, a common mechanism underlying polyploidization. The identification of significant k-mer and LTR sequence divergence between the two haplotypes of *P. tatei* strongly suggests that this species originated from nonrecent distant hybridization, a phenomenon previously hypothesized but never genomically confirmed in *Philodendron*. This discovery aligns with previous observations of hybrid sterility in *Philodendron*, which has historically limited breeding efforts and necessitated reliance on vegetative propagation [16].

Our phylogenomic reconstruction of the Araceae family provides new evidence for the evolutionary positioning of *Philodendron* and reveals complex karyotype evolution patterns among related species. The identification of five distinct genetic clusters within *Philodendron*, coupled with evidence of extensive gene introgression, underscores the role of hybridization as a major force shaping the genus’s diversity. Based on the classification of five groups (A to E), notable differences in reproductive biology are observed between D and E groups compared to the broad-leaved or narrow-leaved A and B groups. Hybrid offspring often exhibit superior growth rates, traits, and stress resistance compared to their parental lines, highlighting the phenomenon of heterosis. In crops such as rice (*Oryza Sativa*), heterosis has been extensively exploited, significantly improving productivity and economic efficiency [29]. In *Philodendron*, widespread intragroup hybridization (including *P. tatei* cv. Birkin) and hybridization among Groups A, B, and C have been observed. Interestingly, with the exception of the Phs51 individual, no notable hybridization events have been recorded within Groups D and E, particularly in Group D. This suggests the potential existence of reproductive barriers between groups. Previous studies have reported chromosome numbers ranging from 28 to 40 in *Philodendron* species [30]. We hypothesize that chromosome homology facilitates meiotic pairing, while variations in chromosome numbers between groups may limit hybridization events. As a result, hybridization between Groups B and C appears to be more frequent and extensive, while hybridization between Groups A and B is also common. This aligns with the general genetic principle that species with closer genetic relationships are more likely to hybridize. In the meantime, we also detected individuals in the group E, which is the furthest from the core of the *Philodendron* cultivars in terms of genetic distance, containing genetic information from the A group or simultaneously from the other three groups. This indicates that this genetic distance does not completely interrupt the exchange of factors among the genetic clusters. It is possible to create more diverse *Philodendron* cultivars through intercluster hybridization. The genetic introgression analysis suggests that gene flow among *Philodendron* cultivars largely follows the phylogenetic relationships. It is possible that hybridization between nonadjacent phylogenetic groups becomes increasingly constrained with greater genetic distance.

Leaf color is one of the most critical commercial traits in *Philodendron* and the broader Araceae family, which is primarily determined by the dynamics of chlorophyll metabolism in leaves [31]. Therefore, the study of its formation is highly significant for breeding and industry applications. By comparing DEGs and ASEs among individuals with varying leaf colors, we identified a chlorophyll degradation-related gene, *PtSGR1*, which exhibits significant differences in expression. This gene, homologous to the STAYGREEN (SGR1) protein in *A. thaliana*, has two haplotype alleles, *SGR1-A* and *SGR1-B*, both of which play a pivotal role in leaf color formation in ornamental *Philodendron*. The differential promoter activities between *SGR1-B* (Phs20) and *sgr1-B* (Phs01) provide a mechanistic explanation for the phenotypic diversity observed, offering a molecular foundation for future breeding strategies. In addition to the effects of transcriptional regulation and structural variation on the chlorophyll metabolism pathway, we also observed that *Philodendron* exhibits young leaf whitening under prolonged exposure to relatively low temperatures (15°C for 30 days). Furthermore, we validated and explored two mechanisms underlying leaf color formation in *Philodendron*: (i) low-temperature conditions significantly promoted high expression of both *SGR1-A* and *SGR1-B* in *P. tatei*, leading to an albino phenotype in newly emerging leaves. (ii) In light-colored individuals of Group D and light-colored hybrids in Group A (e.g. Phs01), a deletion in the promoter region of the *sgr1-B* allele was identified. This deletion altered upstream transcriptional regulation, driving increased *PtSGR1-B* expression and resulting in a chlorotic phenotype (Fig. 4H). Unfortunately, the inability to achieve gene knockout (or knockdown) in the *Philodendron* cultivars has prevented us from elucidating the mechanism underlying the coordinated action of chlorophyll synthesis and degradation responsible for the yellow–green variegation in leaves. In previous studies, environmental factors such as light intensity [32, 33], temperature [34], and photoperiod [35] could interact with the internal genetic regulation of chlorophyll homeostasis. Analyzing this complex potential coordination between environmental sensing pathways and allelic regulatory networks is also the target that we need to further address. However, we were unable to associate this massive gene flow with the formation of the unique leaf color of *Philodendron* yet.

In conclusion, our work establishes a foundational genomic framework for *Philodendron*, bridging the gap between basic research and applied horticulture. Moreover, it traces the footprint of hybridization in shaping species diversity and identify genetic mechanisms underlying leaf color variation. The identification of hybridization-driven diversification and the elucidation of leaf color regulation mechanisms open new avenues for crop improvement, while the genomic resources developed here will serve as a valuable tool for future studies in plant evolution and biotechnology. Future directions should include functional validation of additional candidate genes associated with ornamental traits and the application of genome editing technologies to accelerate cultivar development.

## Method and material

### Plant materials

Samples used for whole-genome and transcriptome sequencing were derived from *Philodendron* species cultivated at the Agricultural Science Research Center of Dongguan, Guangdong, China (E113.76°, N22.95°). Genomic DNA, extracted via a CTAB-based protocol, supported both short-read (MGI platform) and long-read (ONT and PacBio HiFi) sequencing.

### Haplotype-solved genome assembly and annotation

Pacbio and ONT library construction and sequencing were conducted by Novogene (Beijing, China), and Berry Genomics (Beijing, China), respectively. Nanopore sequencing yielded 52.40 Gb of ultralong reads, while 96.97 Gb of HiFi reads were obtained from PacBio Revio. Initial genome assembly for *P. tatei* was performed with HiFiasm, integrating both long-read types [36]. Chromosomal scaffolding was achieved through Hi-C data processed using Juicer and 3D-DNA [37, 38], and refined with Juicebox [39]. An independent ONT-only assembly was generated via NextDenovo and Hifiasm [40], which was subsequently used to close gaps in the chromosomal scaffolds. SubPhaser partitioned haplotypes and characterized specific LTR and K-mer features based on Hi-C linkage signals [18].

Repetitive sequences were annotated using EDTA with default parameters, and subsequently soft-masked [41]. Structural gene prediction on the masked genome was carried out using Helixer, a deep-learning-based tool [42]. RNA-seq data (~30.5 Gb) were aligned using HISAT2 and processed via SAMtools and StringTie to reconstruct 69 212 transcripts [43–45], which were employed by PASA to refine gene models [46].

Assembly quality was evaluated using multiple metrics. Scaffold N50 was calculated to reflect contiguity, and genome completeness was validated through N50 mapping rate, QV value, LAI and BUSCO analysis using the embryophyta_odb10 database [47–49]. Telomeric and gap structures were assessed via querTeT [50].

### Hi-C assays

Hi-C library construction and sequencing was carried out by Qixun Biotech (Nanjing, China). Hi-C technology was utilized to explore the spatial positional relationship of chromatin DNA in the genome. DpnII was used for digestion, with other experimental details following our previous studies. Raw sequencing data were processed to obtain clean data by removing overlapping and low-quality sequences. Reads failing unique alignment to the reference genome at both ends, invalid pairs (e.g. self-loops, edge dangling structures), and PCR amplification duplicates were removed using HiCUP software [51].

### Karyotype experiment

Karyotype experiments followed adapted protocols from previous research [52]. Fresh root tips (5–10 mm) from germinated plants on moist filter paper were pretreated in 2 mM 8-hydroxyquinoline at 4°C for 2 hours, fixed in 45% acetic acid at ~2°C for 10 minutes, and macerated in a 1:1 mixture of 1 mol/L hydrochloric acid and 45% acetic acid at 60°C for 20–23 seconds. Samples were then treated with a 1:1:1 mixture of 1% cellulase, pectinase, and snail enzyme at 37°C, followed by DAPI staining for 5 minutes in the dark at room temperature.

### Phylogenetic relationship and karyotype evolution in the Araceae family

The single-copy orthologue genes of 12 species were identified with OrthoFinder [53] and aligned using MAFFT algorithm [54]. Divergence times with fossil evidence between the species were obtained from TimeTree database (http://timetree.org/). Finally, the mcmctree program from PAML was employed to calculate the time of divergence for the assessed species [55, 56]. Gene family expansion and contraction were conducted via the CAFE software [57].

Paralogous genes were identified with wgdi. The ages of whole-genome duplications were estimated assuming a constant rate of synonymous mutation accumulation. The plant average *Ks*/year rate (*r*) in Araceae was inferred via *Ks* distributions of paralogous genes using the formula divergence data = *Ks*/(2 × *r*) [58, 59].This average *Ks*/year rate was subsequently applied to estimate WGD ages for each sampled Araceae species. The inference of AMK-A was accomplished in our previous study (https://github.com/SunPengChuan/Angiosperm-karyotype-evolution/tree/master/Karyotype/AMK-A) [19, 60].

### DNA extract, sequencing, organelle genome assembly, and population variation analysis

Purified genomic DNA samples were sheared into 350 bp fragments to construct short-insert libraries for 2 × 150 bp sequencing on a DNBSEQ-T7 platform. For the assembly of organelle genome, chloroplast genomes were assembled using the GetOrganelle pipeline with the embplant_pt database [52]. For population analysis, approximately 812 Gb of short-read data from 62 *Philodendron* cultivars and three outgroup cultivars from the *Amydrium* and *Thaumatophyllum* genus were utilized. Short-read data were aligned to the reference genome using the mem module of bwa. Phylogenetic trees, based on both whole chloroplast genome alignments and SNP datasets, were constructed with IQ-TREE [61]. The best-fit substitution model was determined using ModelFinder within IQ-TREE, and branch support was assessed with 1000 ultrafast bootstrap replicates. Phylogenetic relationships of cultivars and outgroups were processed with IQ-TREE based on the SNP matrix. Filtering of genetic variants and population clustering via genetic distance analysis were conducted using plink and vcftools [62]. The filtering thresholds included a minor allele frequency (MAF > 0.05) and a missing data proportion (miss <0.01). Subsequent file processing, variation detection, and calculation of Pi and Fst were performed using samtools and bcftools, respectively [63]. Population structure analysis and gene flow identification employed ADMIXTURE, TreeMix, and Dsuite software [64, 65]. Introgression and topology were conducted using genomics_general (https://github.com/simonhmartin/genomics_general) and TWISST [66].

### Total RNA extraction and RT-qPCR

Total RNA was extracted using SteadyPure Plant RNA Extraction Kit (Accurate, China), and cDNA for gene cloning, and RT-qPCR was synthesized by HiScript II Q RT SuperMix for qPCR (+gDNA wiper) (Vazyme, China). The ChamQ SYBR Color qPCR Master Mix (Vazyme, China) was used for a quantitative real-time PCR (RT-qPCR). The 2^-△△CT^ method was employed to analyze the relative expression levels of each gene [67]. The experiments were conducted with three replicates.

### The processing of transcriptome data and identification of different expression genes

The RNA-seq (Illumina NovaSeq 6000) of six *Philodendron* samples with three replicates (~108 Gb in total) were conducted by Novogene (Beijing, China), including RNA qualification and enrichment, library construction, and sequencing. The comparison of RNA-seq data was processed through the Hisat2 pipeline [44]. The software SAMtools was used to sort the data [45]. The statistics of gene expression was achieved by utilizing the software FeatureCounts [68]. The R module DESeq2 was employed for the purpose of data normalization and the identification of differentially expressed genes (DEGs) [69]. The identification of alleles was performed using the software JCVI [70], and the ASE analysis was accomplished with the R package LIMMA (https://bioconductor.org/packages/release/bioc/html/limma.html).

### Gene cloning and plant transformation

PCR amplification and restriction enzyme digestion were employed to clone coding sequence and promoter of *PtSGR1* with Phs01, Phs20, and Phs50 as templates. The primer sequence can be found in Supplementary Table S18. The resulting recombinant plasmid pEAQ was introduced into Agrobacterium strains (GV3101) via heat shock transformation. Four-week-old *N. benthamiana* leaves was selected for injection using *Agrobacterium* resuspended with infection solution. After co-cultivation in the dark, *N. benthamiana* were transferred to a light incubator and further experimental determination was conducted two days later. For the transient transformation of *Philodendron*, the tissue-cultured seedlings of *P. tatei* were selected and, immersed in an inoculation solution containing Agrobacterium strains (EHA105), and perform vacuum infiltration were performed for 15 minutes. After that, the seedlings were subjected to dark treatment for one day, and then transfered to light conditions for cultivation. Three days later, photography recording and sample collection were conducted.

### Promoter transcriptional activity

The 62-SK empty vector was used as effectors, and the LUC initiated by two types of *proPtSGR1* were used as reporter and transferred into the GV3101 (pSoup-p19) responsive cells. Mix effectors and reporters in a ratio of 9:1 and inject them into the leaves of *N. benthamiana*. The LUC and REN values were determined according to the Dual-Luciferase Reporter Assay System (Promega, USA), and the activities of promoters were detected by the LUC/REN ratio. For the LUC imaging experiment, the leaves were photographed using a multifunctional chemiluminescence imager (Bio-Rad, USA). For GUS activity test, the GUS activity of p1391-promoters injected *N. benthamiana* were detected by GUS stain kit (Coolaber, China).

## Supplementary Material

Web_Material_uhag041

## Data Availability

The raw genome and transcriptome sequencing data generated and analyzed in this study have been deposited in the NCBI Sequence Read Archive (SRA) under BioProject accession number PRJNA1294177 and NGDC Genome Sequence Archive (GSA) under BioProject accession number PRJCA052160. The Phylogenetic tree files have been uploaded to the TreeBASE under accession ID 32444. The assembled genome sequences, as well as corresponding genome annotation files, have been made publicly available on Figshare at the following link: https://figshare.com/articles/dataset/Philodendron_genome_assembly_and_annotation/29625863 (10.6084/m9.figshare.29625863). All other relevant data supporting the findings of this study are available from the corresponding author upon reasonable request.
