# Supplementary material for: Hybridization footprint and the mechanism of leaf color differences in Philodendron cultivars
Source: Hortic Res. 2026 Feb 20;13(5):uhag041. doi: 10.1093/hr/uhag041 (PMC13175634; doi:10.1093/hr/uhag041)
Supplement: Web_Material_uhag041 [file web_material_uhag041.zip › 260107-ThehaplotypemdrPH-SP.docx]

**Hybridization pathways and the formation mechanism of leaf color differences in the cultivated** ***Philodendron* varieties**

**Supplemental Figure**

**
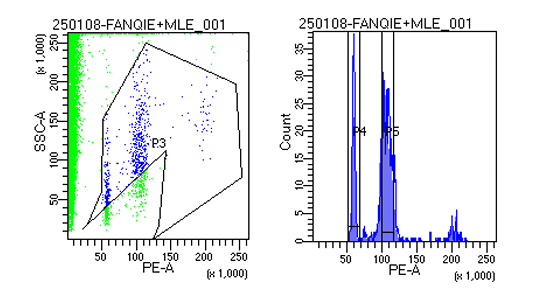
**

**Supplemental Figure 1 The results of estimating the size of genes based on the fluorescence detection of nuclear fluorescence signals using flow cytometry.** Tomato as an internal reference (P4; 1C = 850 Mb); It is estimated that the genome size of *P. tatei* is approximately 1.5 Gb (P5; 1C)


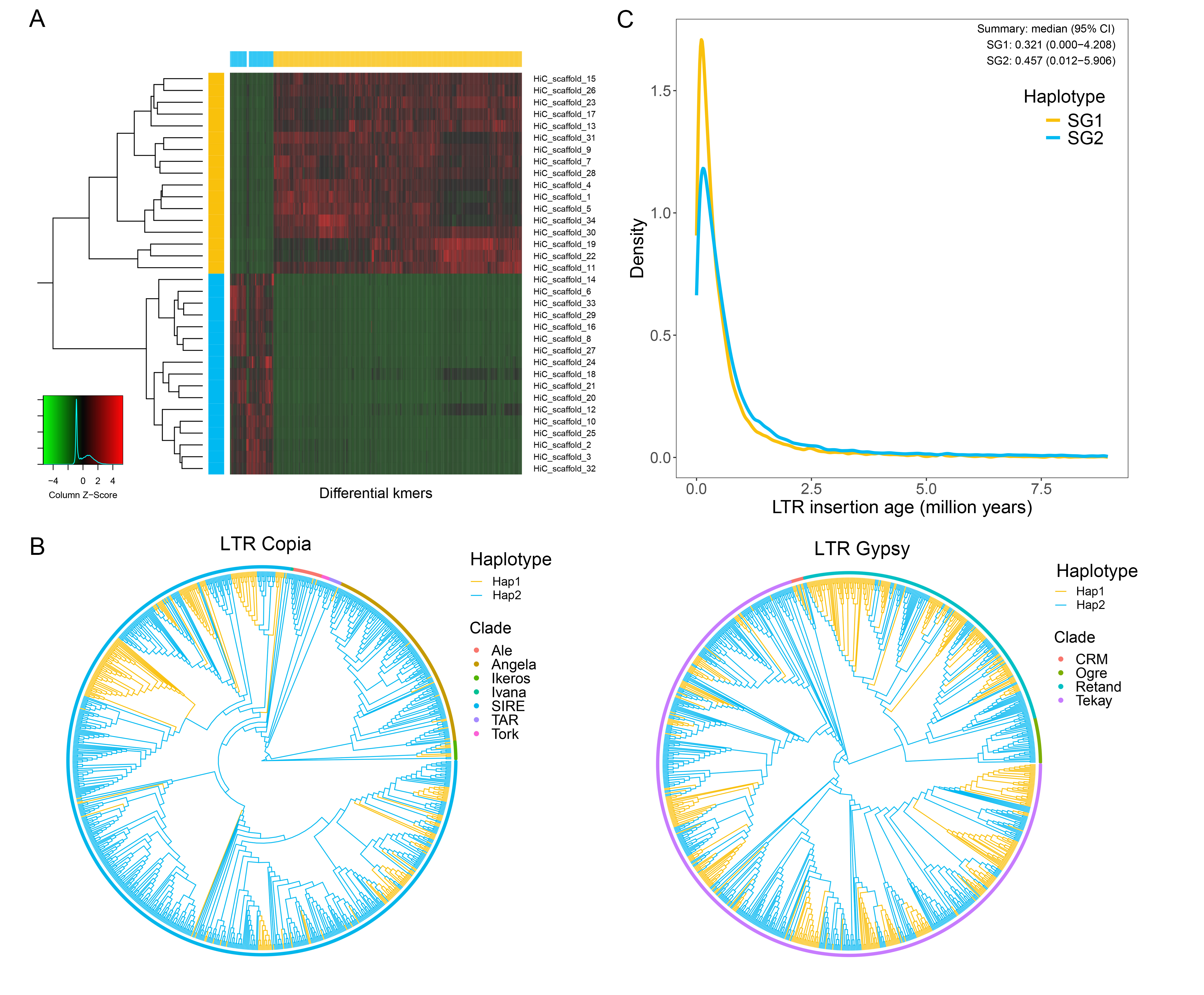


**Supplemental Figure 2**

**Differential LTR distribution among *P. tatei* genome** A: The cluster of the haplotype-specific kmer. B: The phylogenetic tree and evolutionary relationship of the LTR Copia (left) and LTR Gypsy (right) of LTR in the genome. C: The insertion time distribution of specific LTR among haplotypes


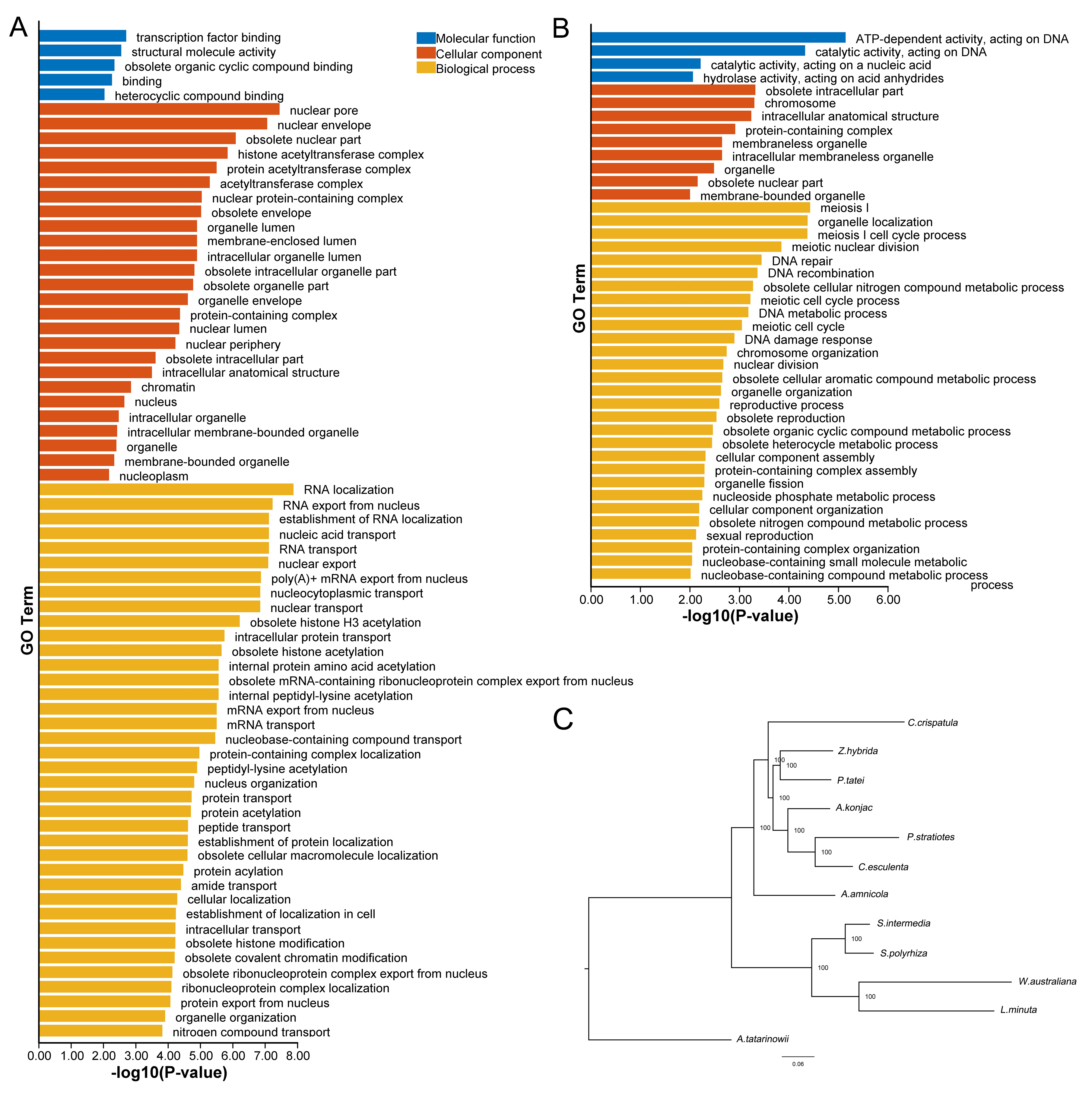


**Supplemental Figure 3**

**GO enrichment of unique genes and phylogenetic relationship of Araceae species.** A-B: GO enrichment of unique genes in HapA (A) and HapB (B) in *P. tatei* genome. C: phylogenetic relationship between *P. tatei* and other Araceae species with bootstrap values (percentage).


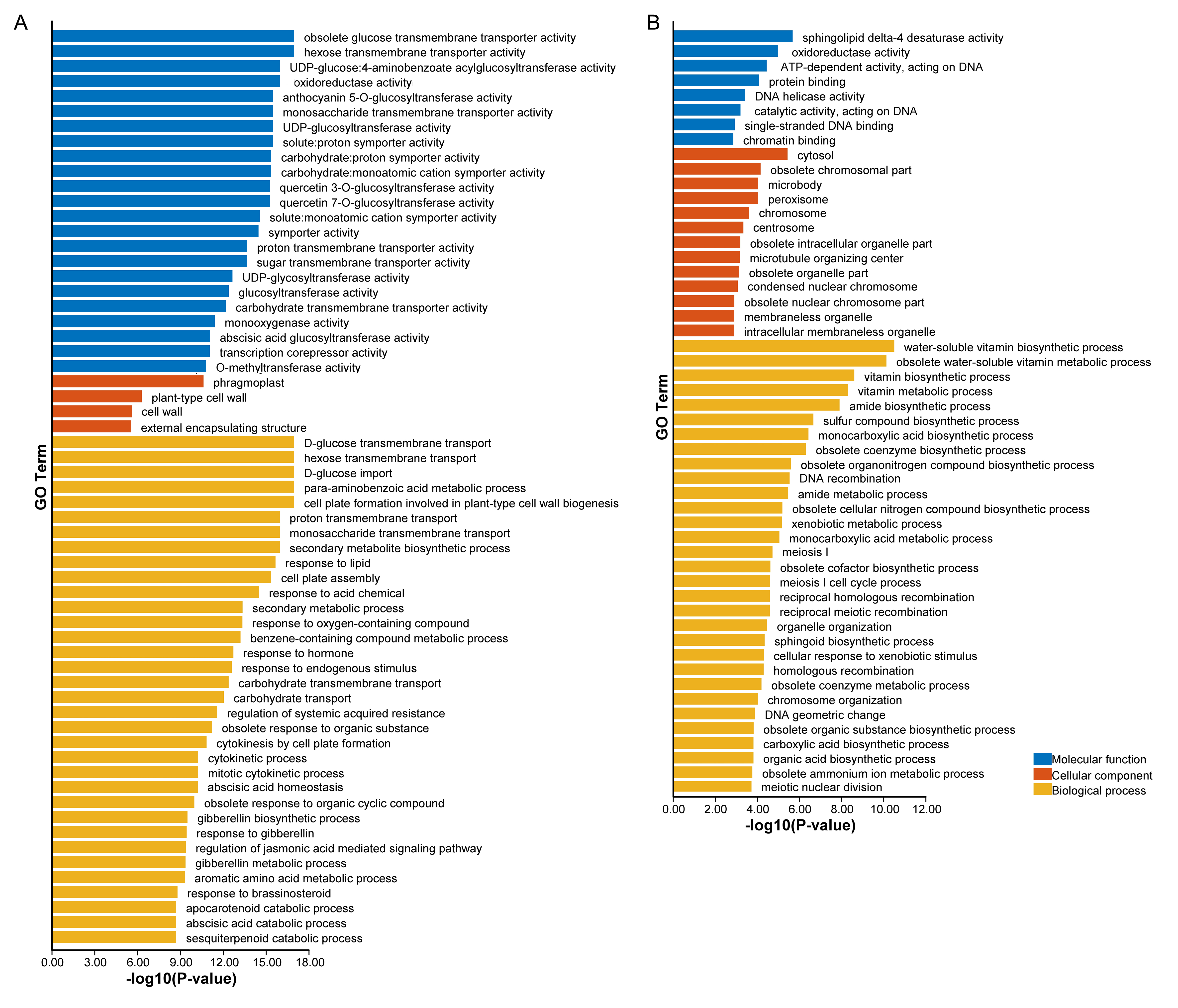


**Supplemental Figure 4**

**GO enrichment of expand (A) and contract (B) genes between *P. tatei* and *Z. hybrida.***


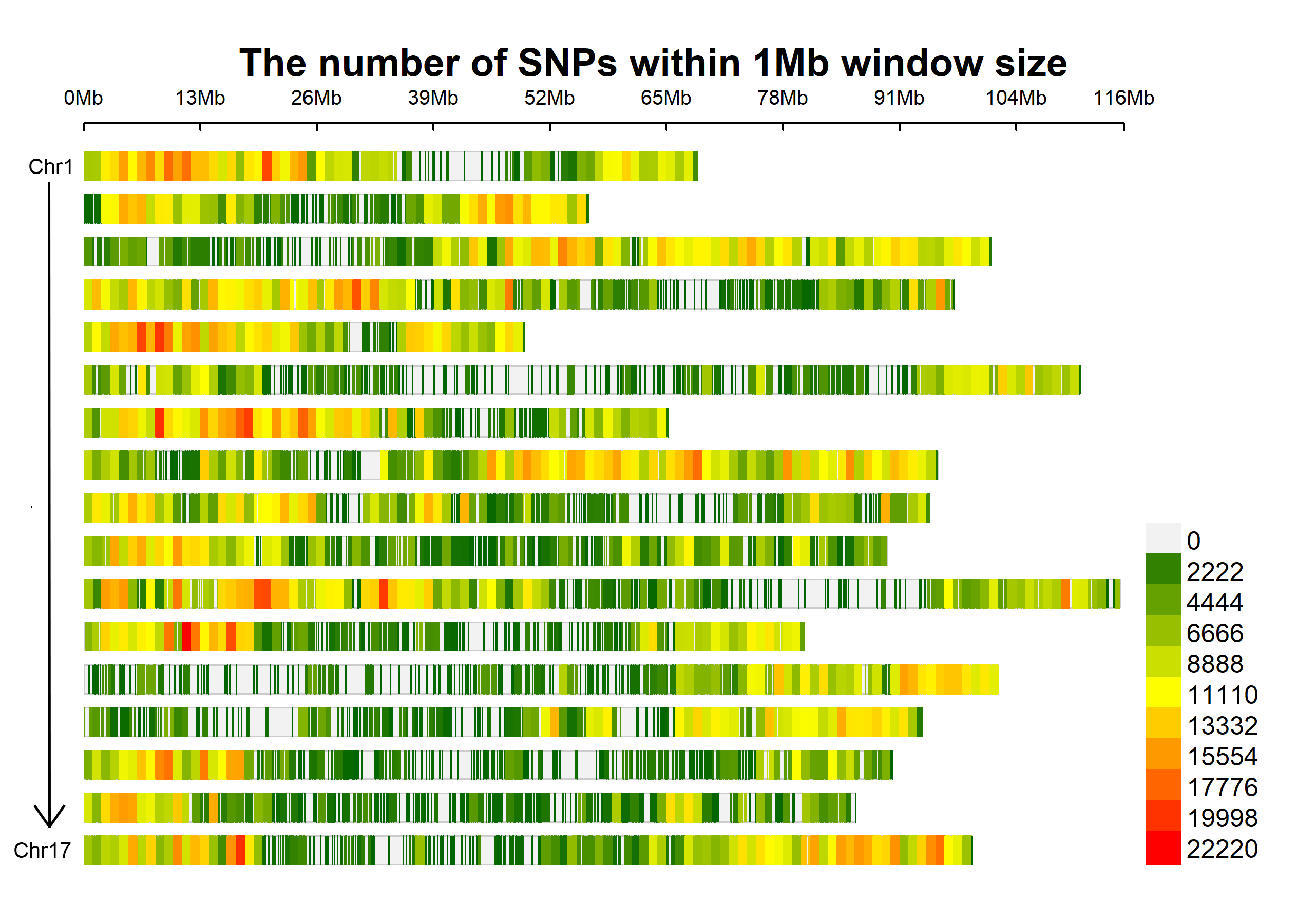


**Supplemental Figure 5**

**The SNP density of *Philodendron* varieties based on the *P. tatei* assembly.**


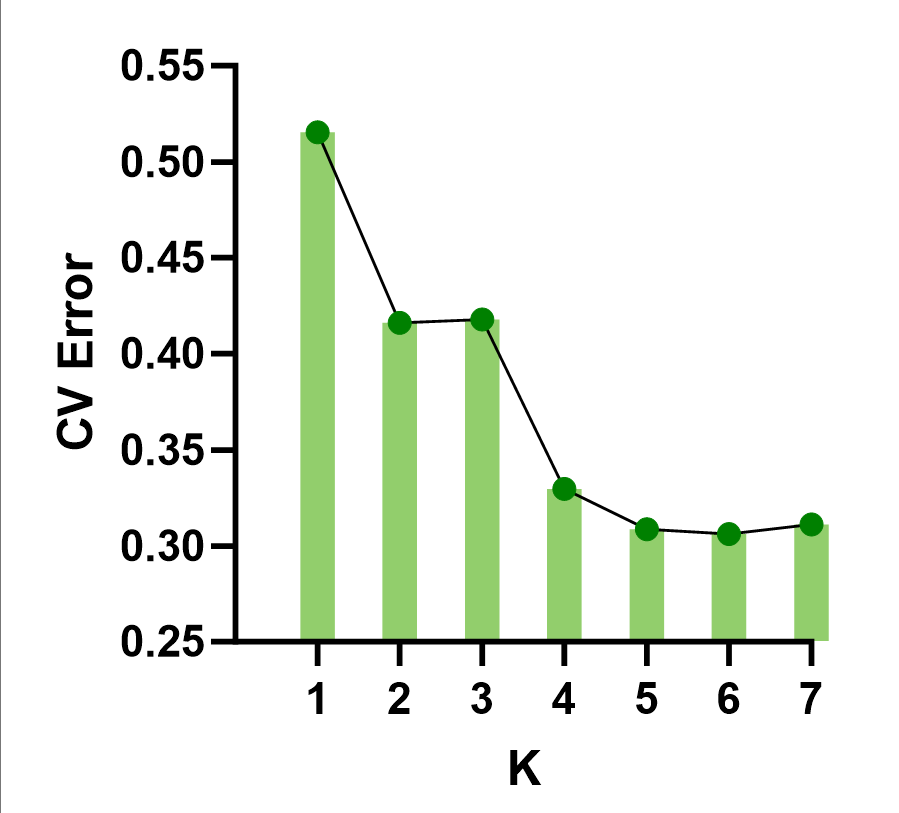


**Supplemental Figure 6**

**Cross-validation (CV) error distribution across different ancestral population numbers (K) in ADMIXTURE analysis of *Philodendron* population.**


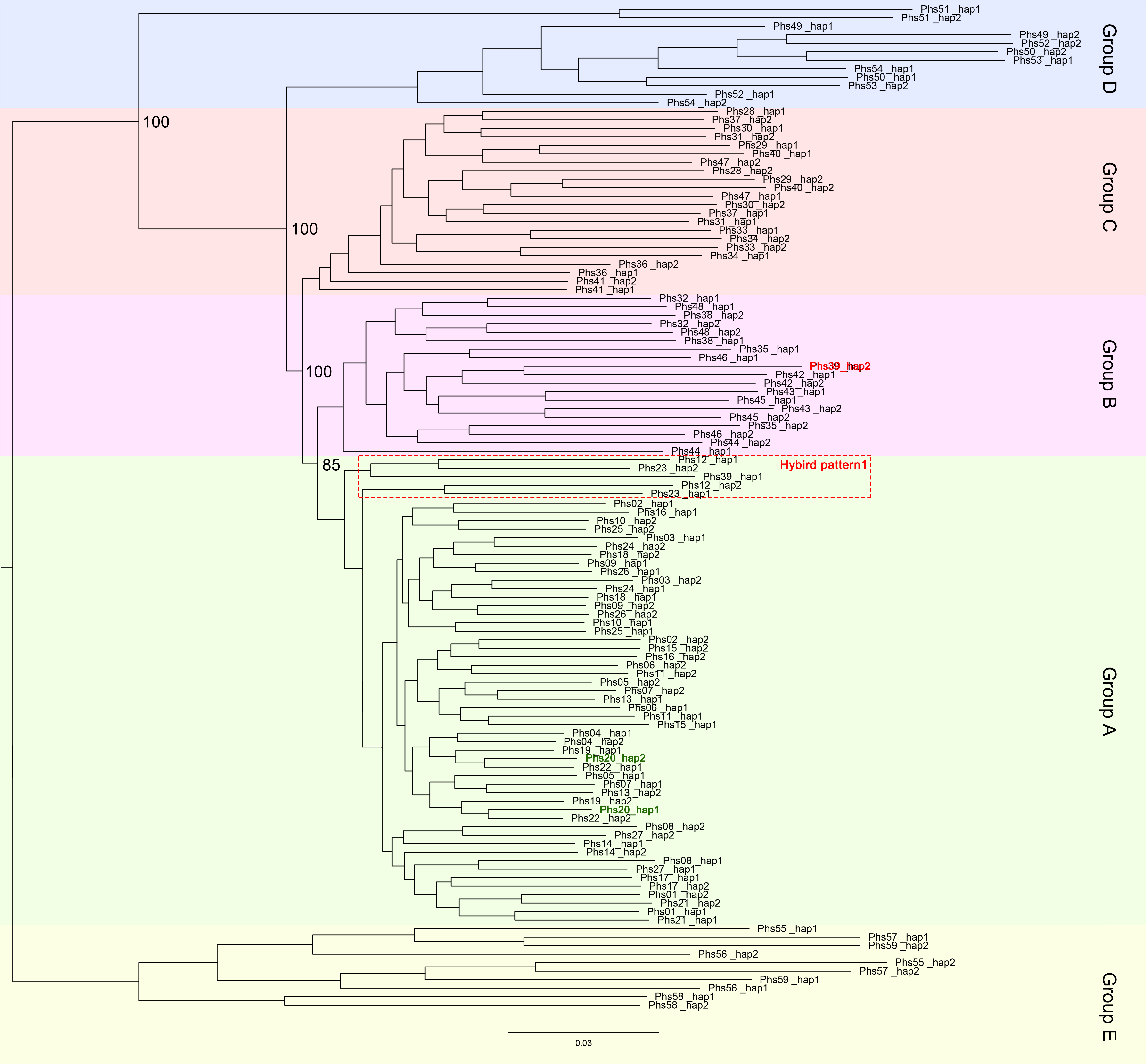


**Supplemental Figure 7**

**Phylogenetic relationships with Maximum Likelihood Tree based on the haplotype genome SNPs among *Philodendron* varieties**


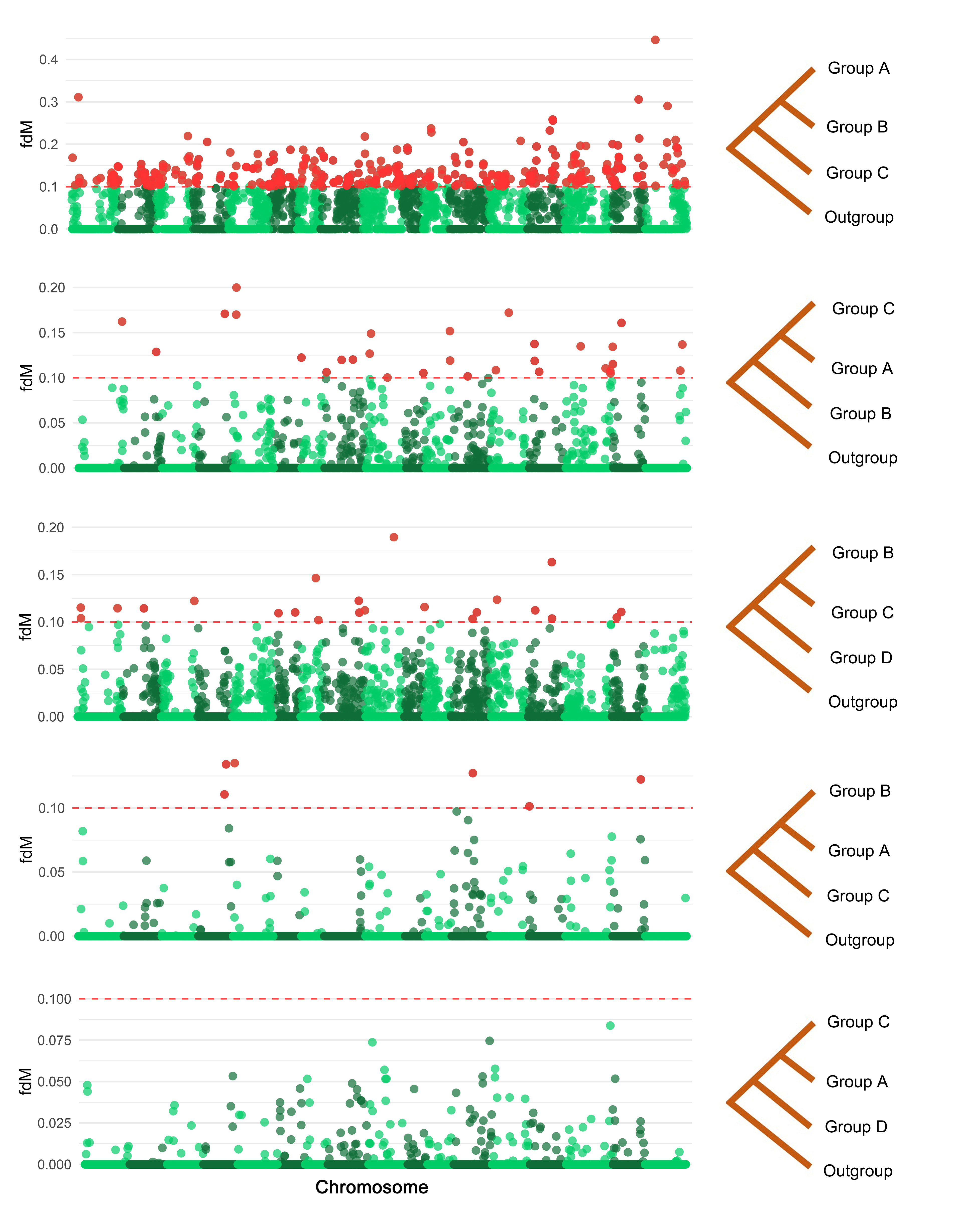


**Supplemental Figure 8**

**Manhattan plot and Patterson’s D statistic of gene flow among different *Philodendron* groups.**

**
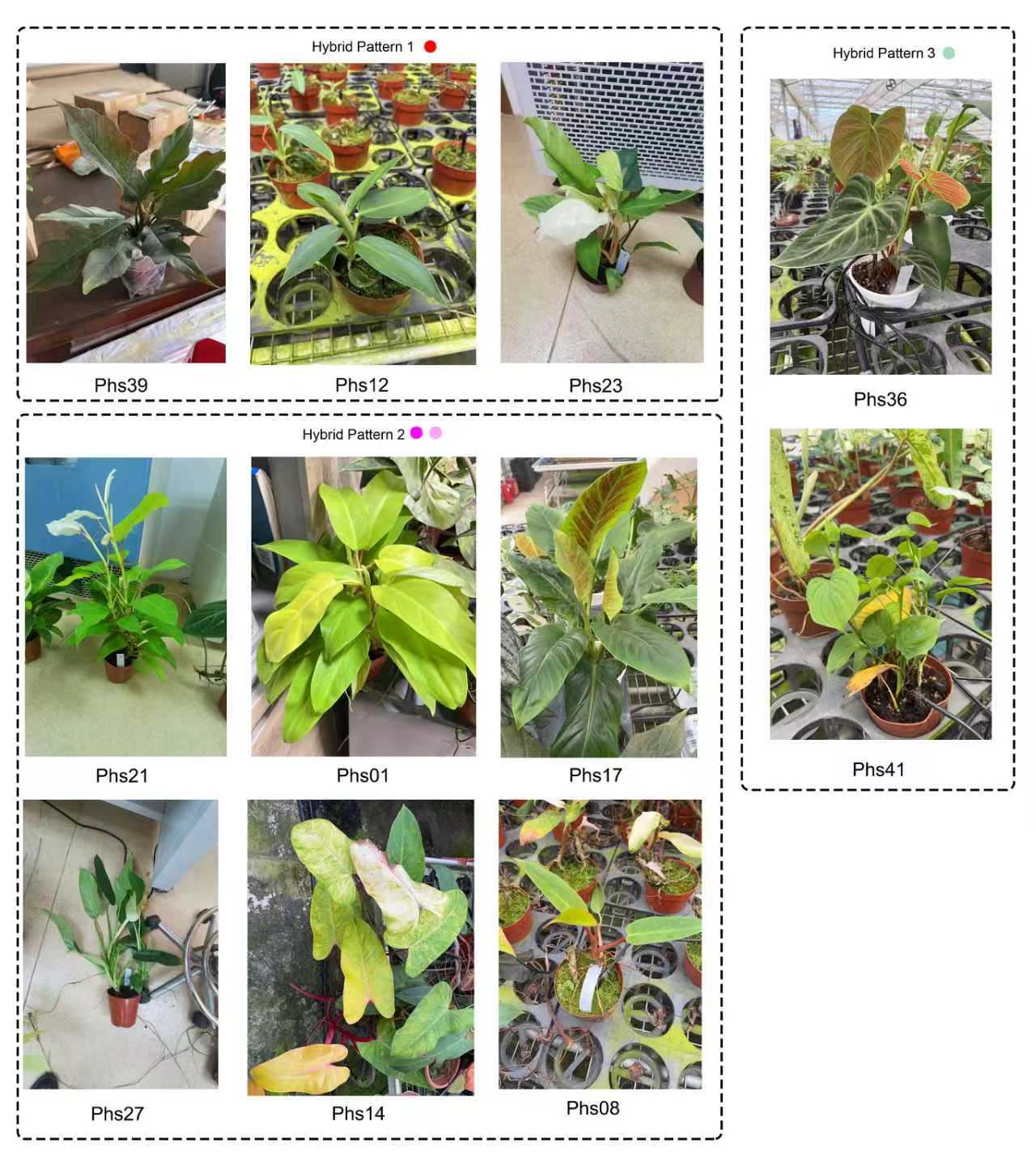
**

**Supplemental Figure 9**

**The phenotype of individual with different hybrid patterns among *Philodendron* varieties.**

^
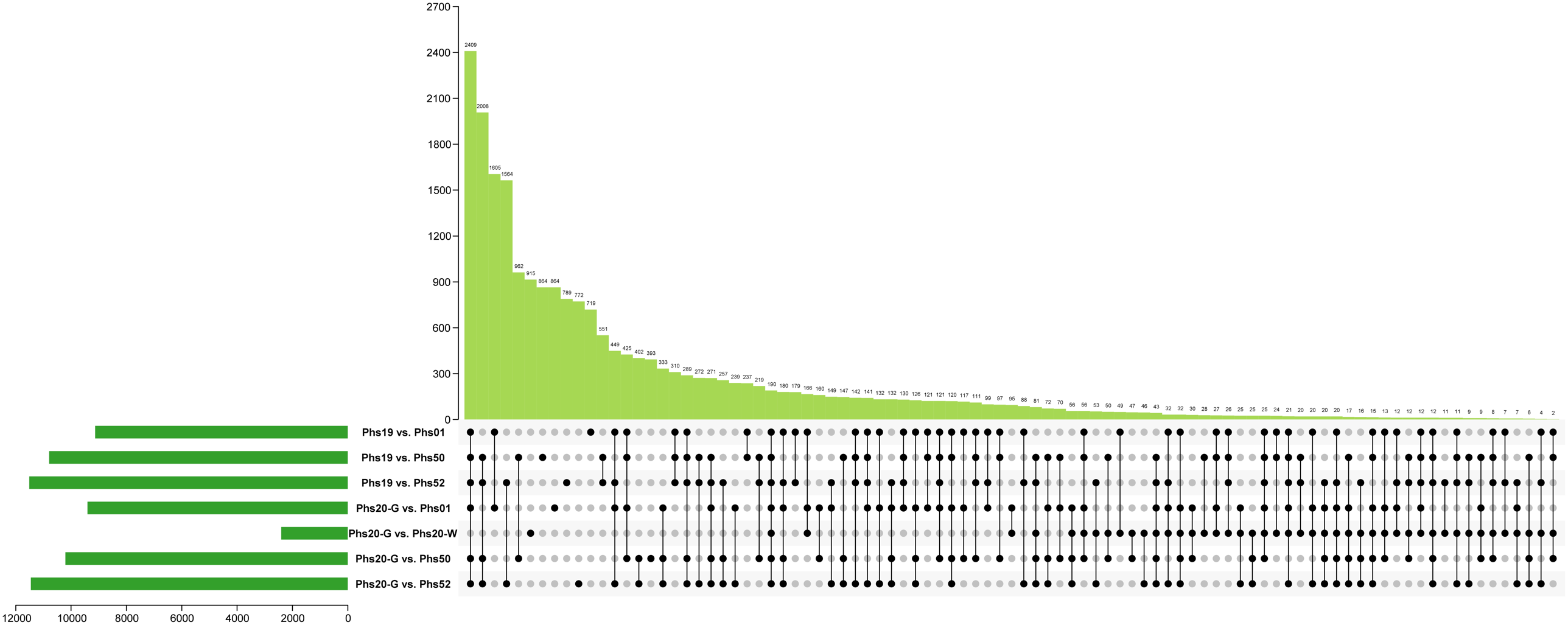
^

**Supplemental Figure 10**

**Upset plot of upregulated-DEGs generated from the transcriptome based on leaves of *Philodendron* varieties.**

^
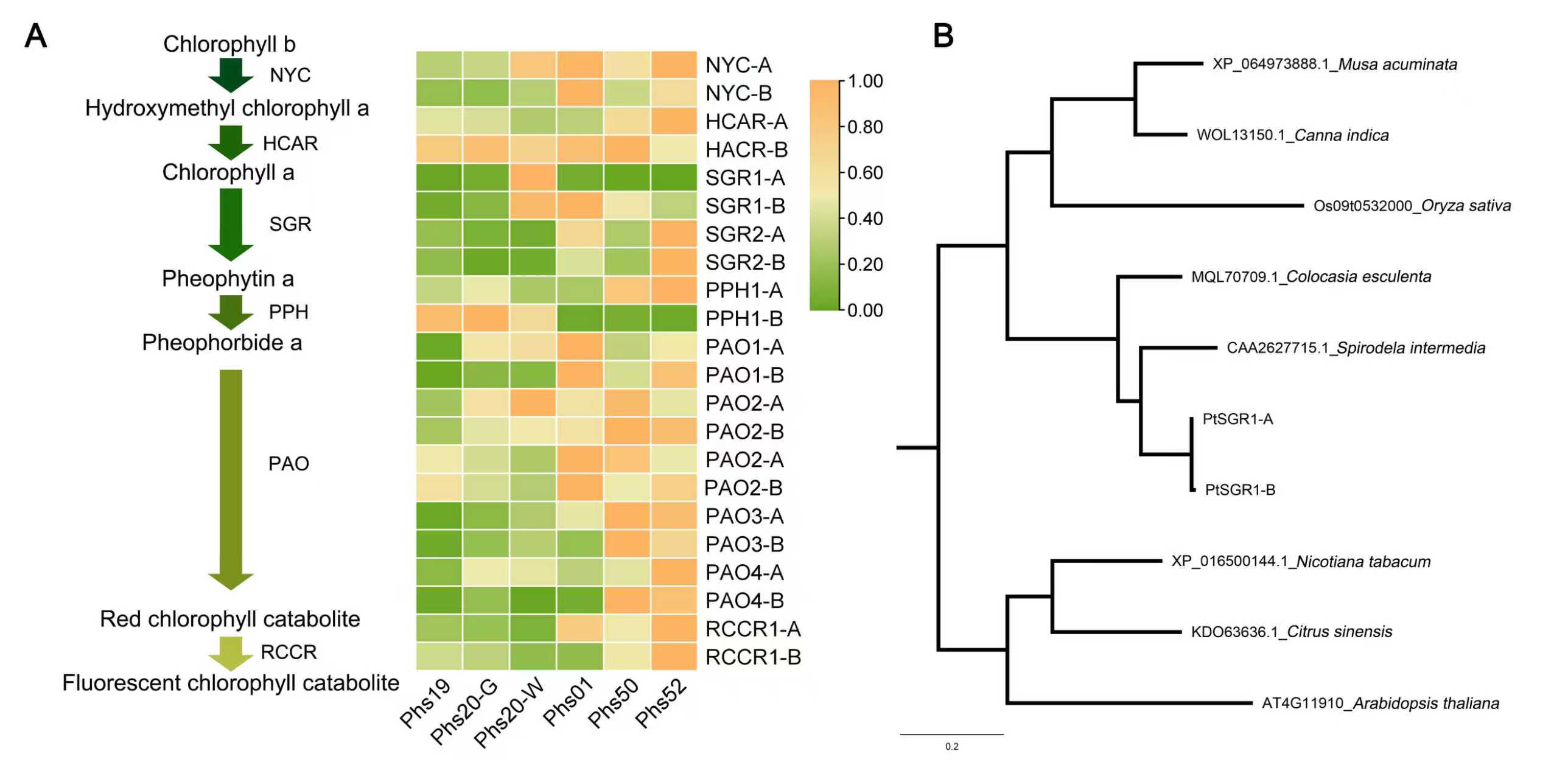
^

**Supplemental Figure 11**

**The pathway of Chlorophyll degradation and the homologous gene of PtSGR1.** (A) Heatmaps displaying the expression patterns of Chlorophyll degradation genes in different leaves of *Philodendron* varieties. (B) Phylogenetic relationships among homologous gene of *SGR1* and two alleles PtSGR1/2.

**
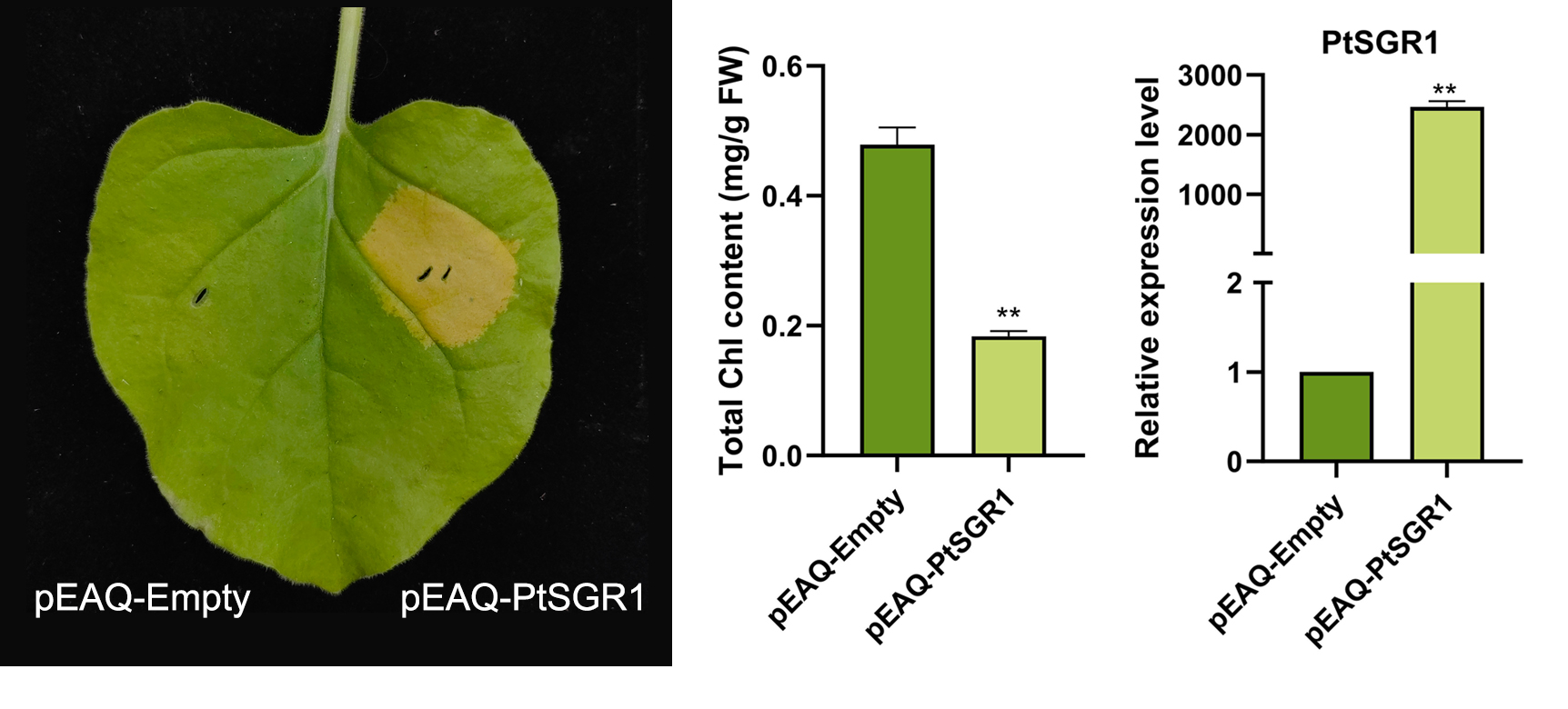
**

**Supplemental Figure 12**

**The transient transformation, Chlorophyll content and expression level of pEAQ-*PtSGR1* with empty vector as control in *N. benthamiana*.**

**
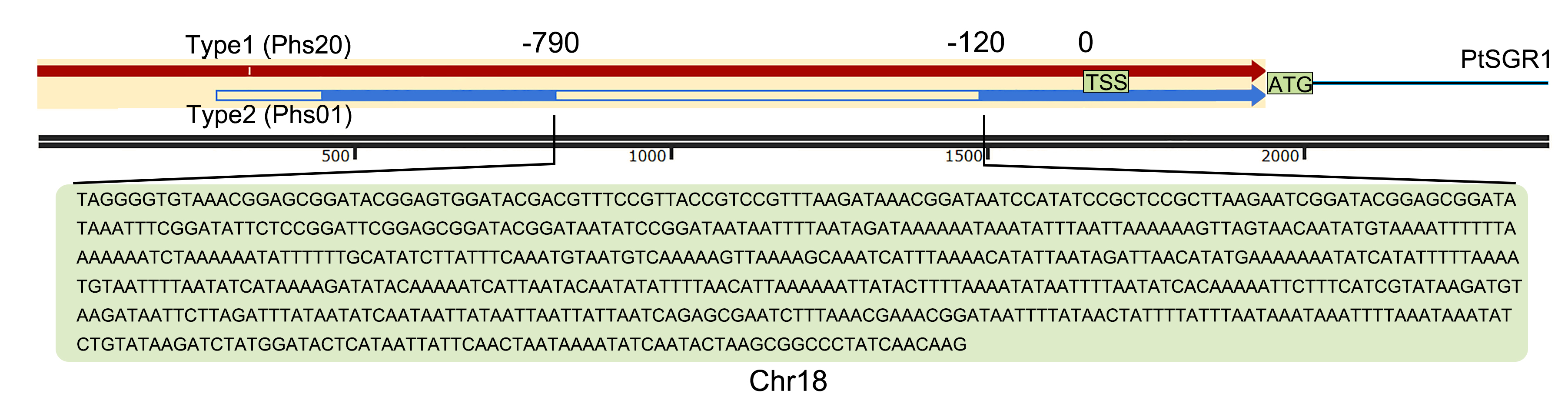
**

**Supplemental Figure 13**

**The structural variation (SV) of PtSGR1-B promoters between Phs20 and Phs01.**


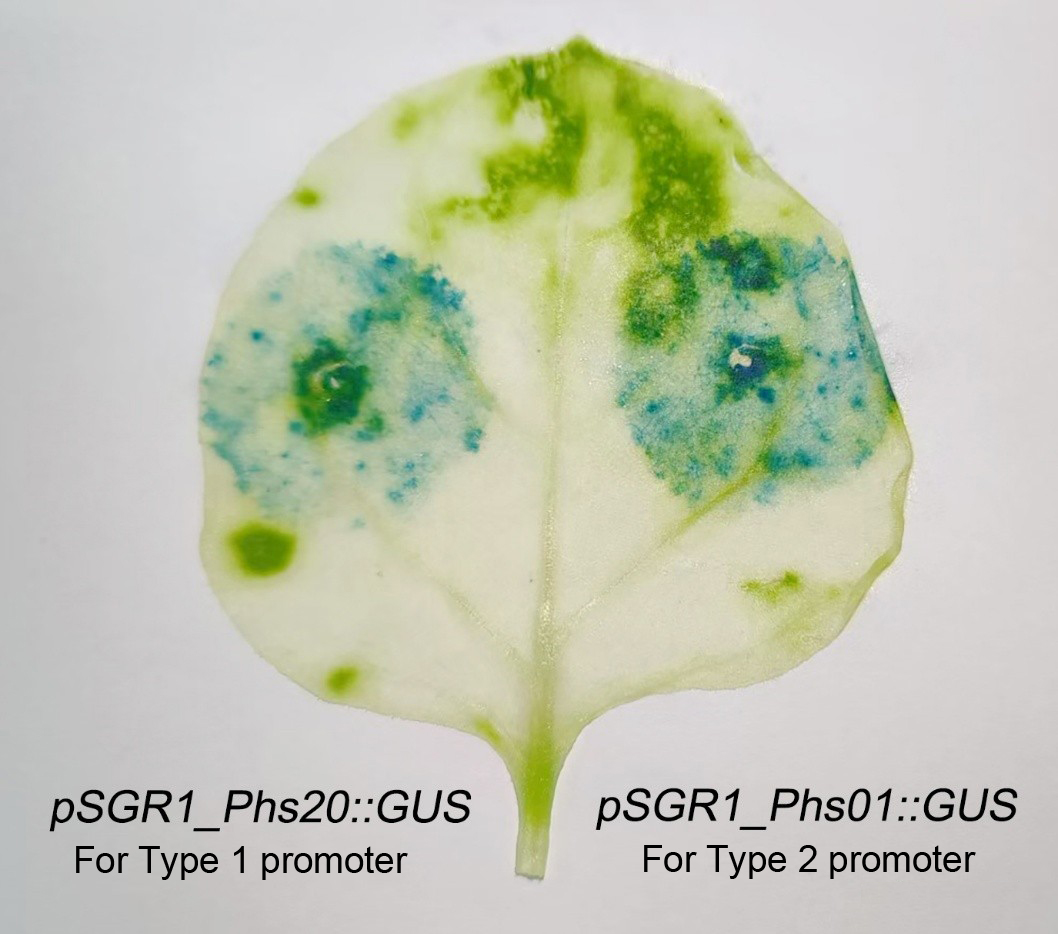


**Supplemental Figure 14**

The GUS staining result of promoter activity assays between two types of *PtSGR1* promoters.
